# Isolation and antisense suppression of *flavonoid 3', 5'-hydroxylase *modifies flower pigments and colour in cyclamen

**DOI:** 10.1186/1471-2229-10-107

**Published:** 2010-06-13

**Authors:** Murray R Boase, David H Lewis, Kevin M Davies, Gayle B Marshall, Deepa Patel, Kathy E Schwinn, Simon C Deroles

**Affiliations:** 1 New Zealand Institute for Plant & Food Research Ltd, Private Bag 11-600, Palmerston North, New Zealand

## Abstract

**Background:**

Cyclamen is a popular and economically significant pot plant crop in several countries. Molecular breeding technologies provide opportunities to metabolically engineer the well-characterized flavonoid biosynthetic pathway for altered anthocyanin profile and hence the colour of the flower. Previously we reported on a genetic transformation system for cyclamen. Our aim in this study was to change pigment profiles and flower colours in cyclamen through the suppression of flavonoid 3', 5'-hydroxylase, an enzyme in the flavonoid pathway that plays a determining role in the colour of anthocyanin pigments.

**Results:**

A full-length cDNA putatively identified as a *F3'5'H *(*CpF3'5'H*) was isolated from cyclamen flower tissue. Amino acid and phylogeny analyses indicated the *CpF3'5'H *encodes a F3'5'H enzyme. Two cultivars of minicyclamen were transformed via *Agrobacterium tumefaciens *with an antisense *CpF3'5'H *construct. Flowers of the transgenic lines showed modified colour and this correlated positively with the loss of endogenous *F3'5'H *transcript. Changes in observed colour were confirmed by colorimeter measurements, with an overall loss in intensity of colour (C) in the transgenic lines and a shift in hue from purple to red/pink in one cultivar. HPLC analysis showed that delphinidin-derived pigment levels were reduced in transgenic lines relative to control lines while the percentage of cyanidin-derived pigments increased. Total anthocyanin concentration was reduced up to 80% in some transgenic lines and a smaller increase in flavonol concentration was recorded. Differences were also seen in the ratio of flavonol types that accumulated.

**Conclusion:**

To our knowledge this is the first report of genetic modification of the anthocyanin pathway in the commercially important species cyclamen. The effects of suppressing a key enzyme, F3'5'H, were wide ranging, extending from anthocyanins to other branches of the flavonoid pathway. The results illustrate the complexity involved in modifying a biosynthetic pathway with multiple branch points to different end products and provides important information for future flower colour modification experiments in cyclamen.

## Background

**Figure 1:**
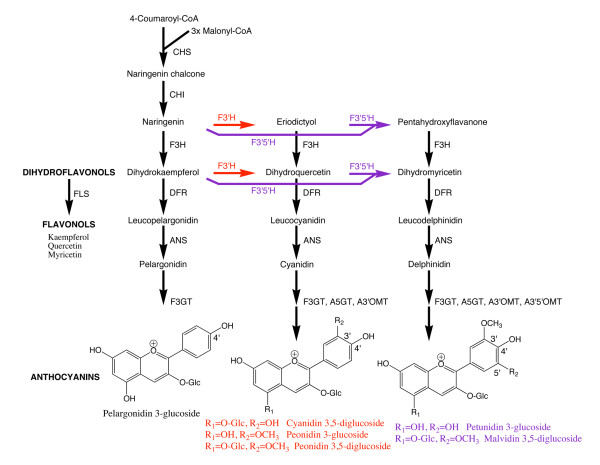
**A simplified version of a section of the flavonoid biosynthetic pathway**. The flavonols kaempferol, quercetin and myricetin are formed from dihydrokaempferol, dihydroquercetin and dihydromyricetin, respectively, by flavonol synthase (FLS). The double arrows show the points of possible action of multiple enzymes for formation and modification of the anthocyanins. Abbreviations are as follows: CHS, chalcone synthase; CHI, chalcone isomerase; F3H, flavanone 3-hydroxylase; DFR, dihydroflavonol 4-reductase; ANS, anthocyanidin synthase; F3'H, flavonoid 3'-hydroxylase; F3'5'H, flavonoid 3',5'-hydroxylase; F3GT, flavonoid 3-*O*-glucosyltransferase; A5GT, anthocyanin 5-*O*-glucosyltransferase; A3'OMT, anthocyanin 3'-*O*-methyltransferase; A3'5'OMT, anthocyanin 3',5'-*O*-methyltransferase. The numbering of the 3', 4' and 5' carbon positions is shown on the anthocyanin structure.

*Cyclamen persicum* Mill. (cyclamen) is a popular and economically significant pot plant crop in Japan, Germany, Italy, the Netherlands and North America. Flower colour in commercial lines ranges from white, through red, pink, reddish-purple to purple. The pigments present are predominantly anthocyanins and there have been several studies on anthocyanin and flavonoid pigmentation in cyclamen [1-5]. The main anthocyanins are 3,5-di-*O*-glucosides of peonidin, cyanidin and malvidin (Figure. 1). There are two missing colour groups in cyclamen, the orange-red of pelargonidin-derived anthocyanins [6] and blue, even though some delphinidin-derived anthocyanins often associated with blue flower colours are present in maroon to purple cultivars [1-3,6].

To date there has only been one reported molecular breeding experiment involving flavonoid pigments for cyclamen. It was focused on the generation of yellow flower colours through the production of yellow flavonoid pigments [7]. Our interest is in altering the anthocyanin-based colours [8]. In flower colour modification studies in general, particular attention has been paid to the enzymes responsible for the hydroxylation of the B-ring of the flavonoid molecule, namely F3'H and F3'5'H (Figure. 1) because of their key influence on the colour of anthocyanin pigments [9]. Specific experiments to accumulate delphinidin-derived anthocyanins by over expression of a *F3'5'H *transgene have been reported for carnation [10] and rose [11], while inhibition of both the *F3'H *and the *F3'5'H *genes has been used to modify colour and promote cyanidin- and pelargonidin-based pigment accumulation in flowers in the genera *Torenia *[12], *Nierembergia *[13] and *Osteospermum *[14].

Our strategy for modification of flower colour in cyclamen focused on the F3'5'H. Substrate feeding experiments with DHK and the F3'H/F3'5'H inhibitor tetcyclacis indicate that the cyclamen DFR can use DHK and that cyclamen has the ability to make pelargonidin-derived anthocyanins (K. Schwinn, unpublished data). The cloning of a *F3'5'H *cDNA and our cyclamen genetic transformation system [15] have allowed us to investigate flower colour formation in cyclamen. In this study we report on the effects of antisense suppression of *F3'5'H *on flavonoid end-product accumulation and flower colour.

## Results

### Isolation and sequence analysis of a cyclamen flavonoid 3', 5'-hydroxylase cDNA

A putative full-length cDNA for *F3'5'H *(*CpF3'5'H*) was isolated from a cDNA library made from mixed flower bud stages of *C. persicum *'Sierra Rose'. The complete nucleotide sequence has 1719 nucleotides with a single major ORF encoding 508 amino acid residues (GenBank accession GQ891056).

**Figure 2:**
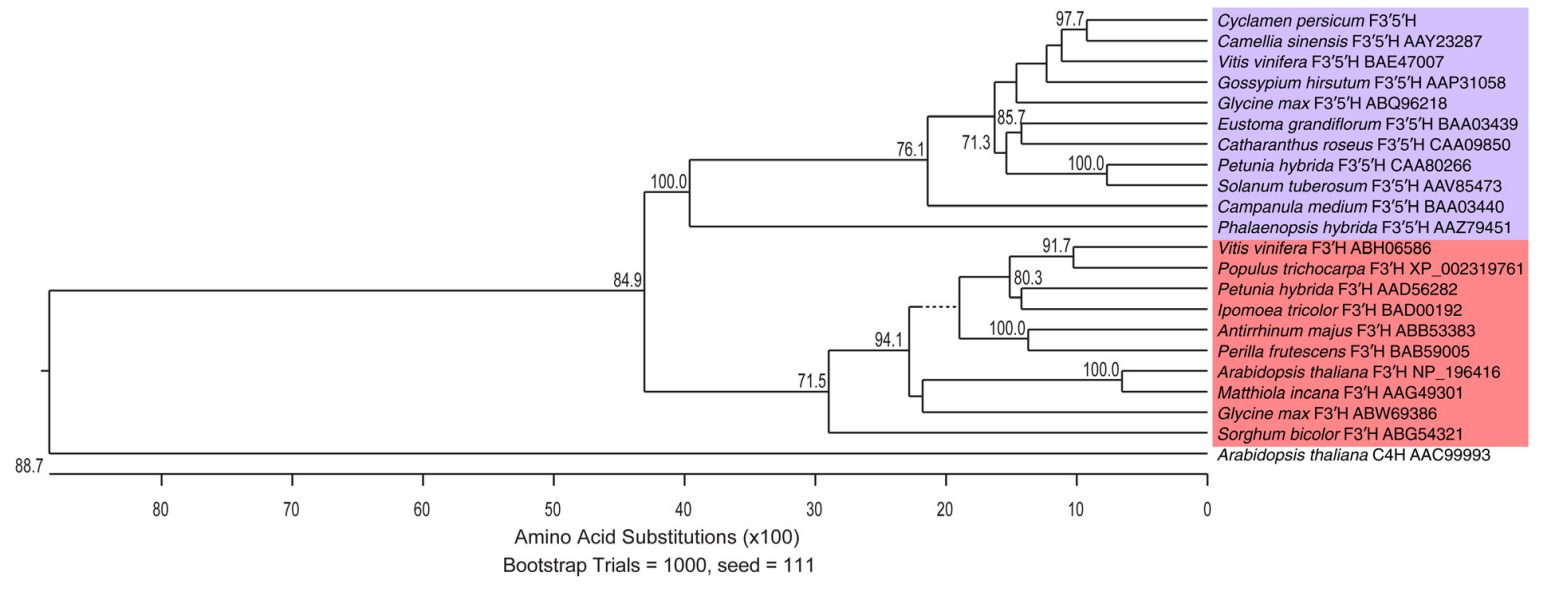
**A phylogenetic tree inferred using CLUSTAL W from the deduced amino acid sequences for *F3'5'H*, *F3'H and C4H***. *F3'5'H *(mauve shading), *F3'H *(red shading), *C4H *(cinnamate 4-hydroxylase, no shading), a less closely related sequence for a cytochrome P450 enzyme involved in flavonoid biosynthesis. The phylogenetic tree shows bootstrap values. The *F3'5'H *sequences form a distinct cluster, which includes the cyclamen sequence. Based on the amino acid and phylogeny analysis the evidence supports *CpF3'5'H *as encoding *F3'5'H*.

When the deduced amino acid sequence for *CpF3'5'H *was used in a BLAST search of GenBank http://www.ncbi.nih.gov/blast/, the closest sequence was the putative *F3'5'H *from *Camellia sinensis *(GenBank accession AAY23287), with 83% amino acid identity. The Lasergene program MegAlign (DNASTAR Inc., Madison, USA) was used to compare the *CpF3'5'H *deduced amino acid sequence with ten *F3'5'H *sequences (the CYP75A group), ten *F3'H *sequences (CYP75B) and two 'outlier' cytochrome P450 sequences (data not shown). Amino acid identity of *CpF3'5'H *to other *F3'5'H *sequences was in the range from 75-82%, except for the *Campanula medium F3'5'H *sequence (BAA03440), 68% identity, which is suggested to have a distinct *F3'5'H *structure [16] and the monocot *Phalaenopsis hybrida F3'5'H *sequence (AAZ79451, 50% identity) [17]. A phylogenetic tree was formed using the CLUSTAL W algorithm http://www-bimas.cit.nih.gov/clustalw/clustalw.html with the MegAlign data (Figure. 2). The *F3'5'H *sequences form a distinct cluster, which includes the cyclamen sequence. Based on the amino acid and phylogeny analysis the evidence supports *CpF3'5'H *as encoding a F3'5'H enzyme.

### Generation of transformed lines and transgene expression analyses

**Figure 3:**
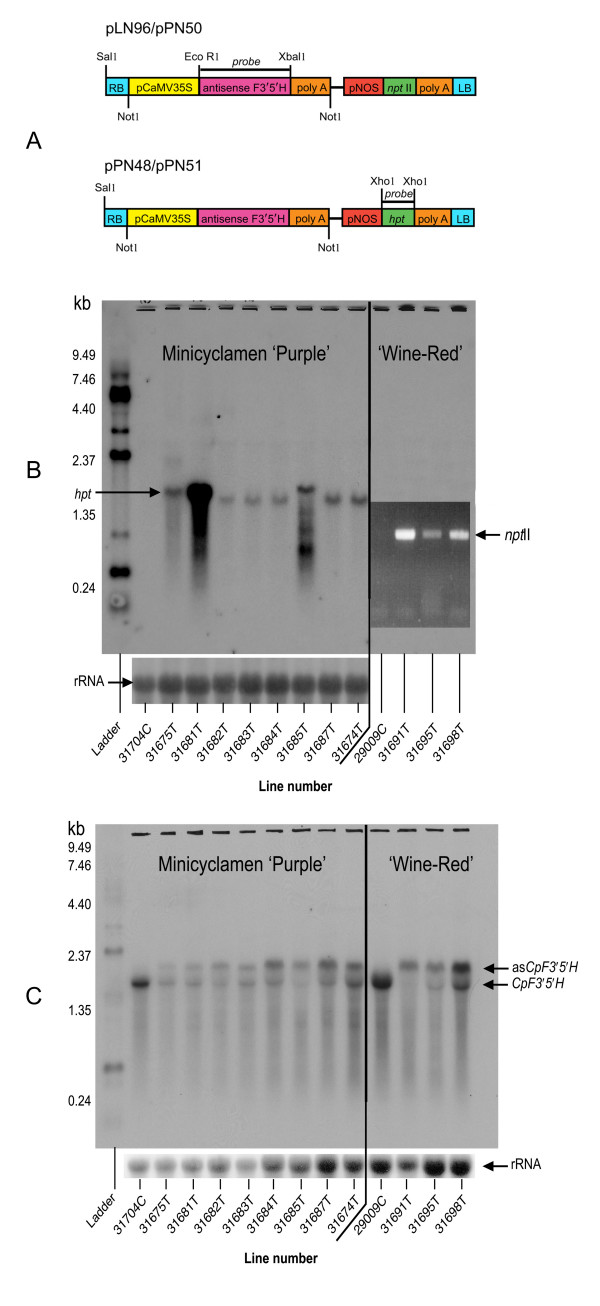
**Northern analysis of Minicyclamen transgenic lines**. A) Schematic diagrams of the T-DNA regions of binary vectors, pLN96, pPN48, pPN50 or pPN51. These binary vectors harboured in their T-DNAs the cyclamen antisense *F3'5'H *gene under a CaMV35S promoter and either *npt*II or *hpt *selectable marker genes under a NOS promoter. B) Northern RNA blot analysis of *hpt *selectable marker expression in control and transgenic lines of cv 'Purple' (left) and RT-PCR analysis of *npt*II selectable marker expression of cv 'Wine-Red' (right). The expected size of the *hpt *signal was 1.4kb and the expected size of the *npt*II signal was 600bp. C) Northern RNA blot analysis of sense and antisense *CpF3'5'H *transcript in transgenic and control lines of cv 'Purple' (left) and cv 'Wine-Red' (right).

**Figure 4:**
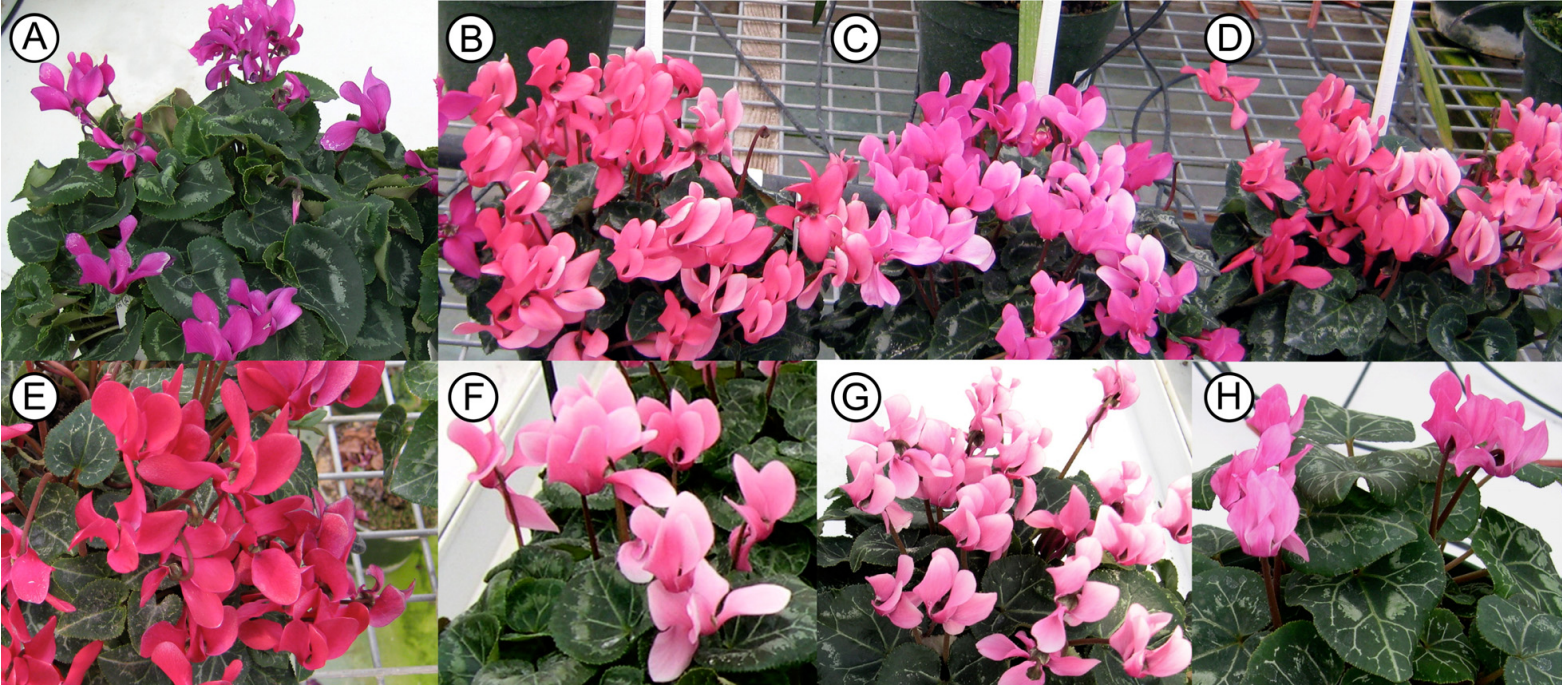
**Flower colour phenotypes of selected transgenic lines**. Cv 'Purple' (A-D); A-#31704 regeneration control line, B-#31674, C-#31682, D-#31683, antisense *CpF3'5'H *transgenic lines. Cv 'Wine-Red' (E-H): E-#29009 regeneration control line; F-#31691, G-#31695, H-#31698, antisense *CpF3'5'H *transgenic lines.

Antisense *CpF3'5'H *transformants were produced from the 'Purple' cultivar using constructs pPN48/51, and from the 'Wine-Red' cultivar using pLN96/pPN50 (Figure. 3A). Flowers from several of the transgenic lines showed significant changes in colour, both in hue and intensity (chroma) (Figure. 4). No other phenotypic alterations were observed when compared with wildtype plants.

Northern blot analysis of cultivar (cv) 'Purple' transformants showed that eight lines were transgenic for the hygromycin selectable marker (Figure. 3B). RT-PCR analysis of the *npt*II selectable marker showed the three cv 'Wine-Red' lines were also transgenic as expected (Figure. 3B).

Northern blot analysis with a mixed sense and antisense *CpF3'5'H *probe, (1.7 kb *Xba*I-*Eco*RI fragment, Figure. 3A), showed that two *F3'5'H *specific transcripts were detected (Figure. 3C). There was a marked reduction in endogenous *CpF3'5'H *transcript in all antisense lines of both cultivars. Antisense *CpF3'5'H *transcript was detected only in the transgenic lines and the levels varied between lines.

### Flavonoid analyses

**Figure 5:**
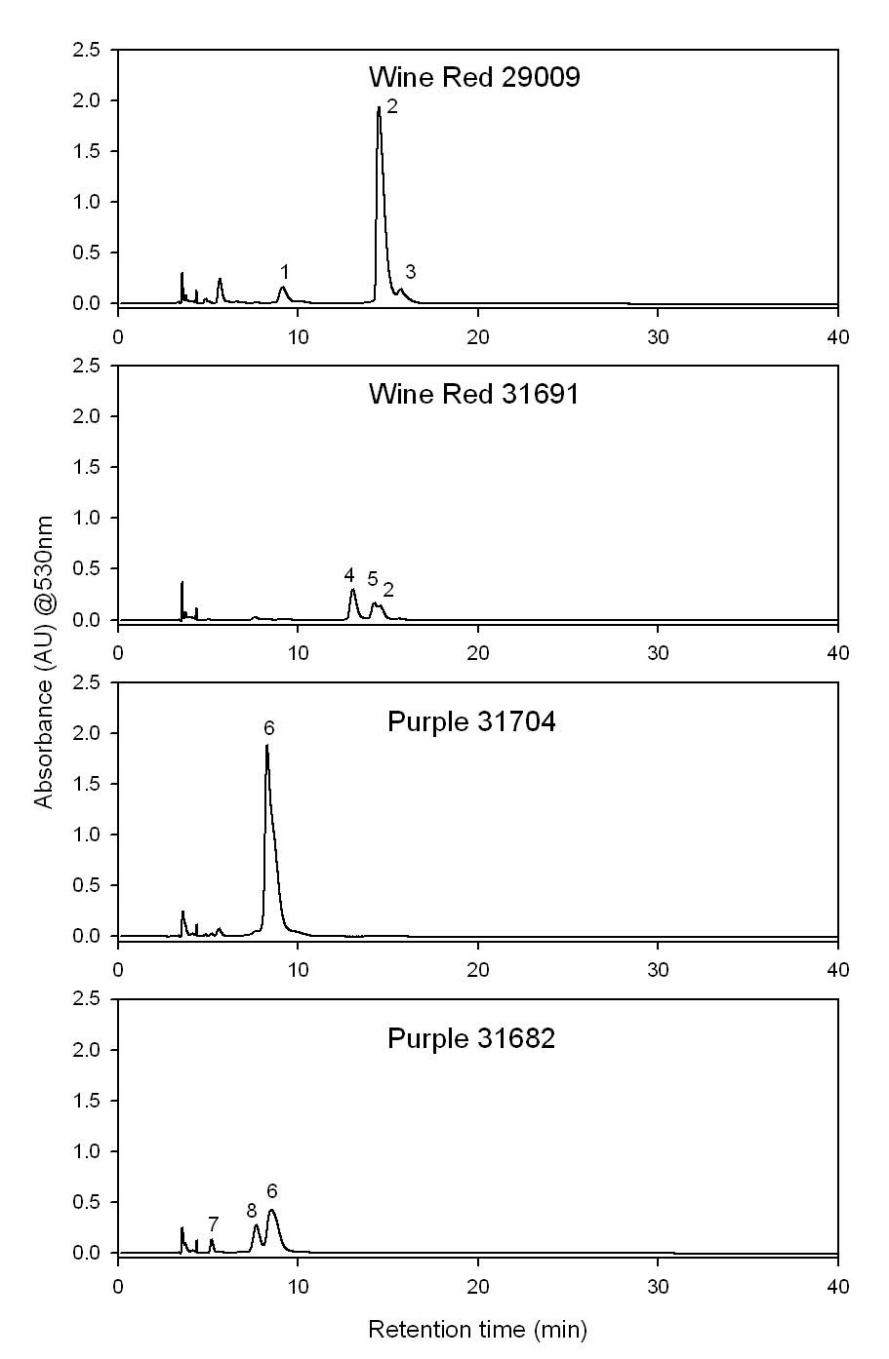
**HPLC chromatograms for petal extracts from selected transgenic lines**. Untransformed control lines were #29009 and #31704. Absorbance was monitored at 530 nm. The major anthocyanins were identified as: (1) Petunidin 3-*O*-glucoside; (2) Malvidin 3-*O*-glucoside; (3) Malvidin rhamnosyl-glucoside; (4) Peonidin 3- *O *-glucoside; (5) peonidin rhamnosyl-glucoside; (6) Malvidin 3-5-di-*O*-diglucoside; (7) Cyanidin 3-5-di-*O*-diglucoside; (8) Peonidin 3-5-di-*O*-diglucoside.

**Figure 6:**
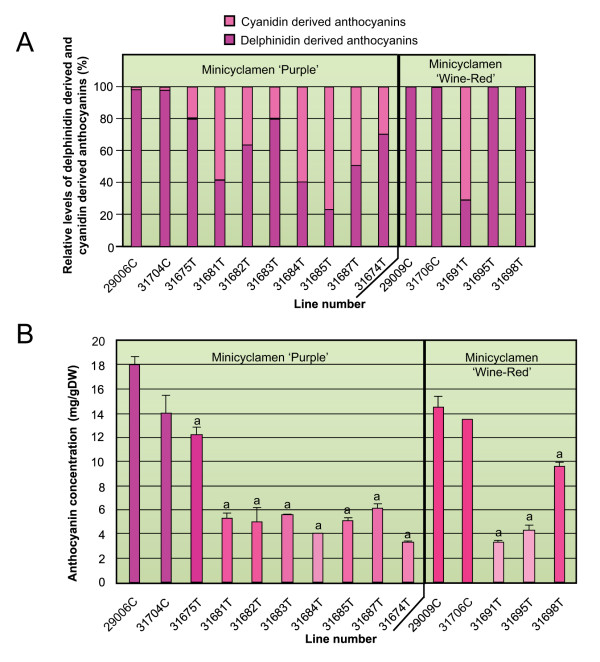
**Anthocyanin profiles of minicyclamen transgenic flowers**. A) Relative proportions of delphinidin-derived and cyanidin-derived anthocyanins in the flower petals. Control lines are denoted C and transgenic lines by T. B) Total anthocyanin concentrations in the flower petals of transgenic lines. Colour bars are representative of actual petal colour of each line. Control lines are denoted C and transgenic lines by T. Mean ± SEM, n = 2. Values significantly different from the control at the 5% level have been indicated by a superscript ^a^.

Anthocyanin content in the petals of the transgenic lines changed in both concentration and profile. The anthocyanins detected in the flower tissue of the regeneration control plants and transgenic lines are shown in Figure. 5 and 6 and are listed in Table 1. Anthocyanin identities were assigned by retention times and mass spectrometer data and were consistent with the anthocyanins identified previously for cyclamen, predominantly the 3-mono and 3,5 di-glucosides of peonidin, cyanidin and malvidin [2,4]. Malvidin 3*-O*-glucoside was the predominant anthocyanin in cv 'Wine-Red' while malvidin 3,5 di-*O*-glucoside was the predominant anthocyanin in cv 'Purple'.

**Table 1:** HPLC-MS^2 ^based identifications of the main anthocyanins detected in petal tissue.

Peak number	Anthocyanin*	T_r _(min)	λ_max_(nm)	[M]^+ ^(m/z)	MS/MS
1	Petunidin 3-*O*-glucoside;	9.3	277, 343, 526	479	317
2	Malvidin 3-*O*-glucoside;	14.5	277, 348, 531	493	331
3	Malvidin rhamnosyl-glucoside	15.7	277, 348, 531	639	331, 493
4	Peonidin 3-*O*-glucoside	13.0	282, 330, 516	463	301
5	Peonidin rhamnosyl-glucoside	14.2	282, 330, 521	609	301,463
6	Malvidin 3,5-di-*O*-glucoside	8.7	277, 343, 531	655	331, 493
7	Cyanidin 3,5-di-*O*-glucoside	5.2	282, 516	611	287,449
8	Peonidin 3,5-di-*O*-glucoside	7.7	277, 330, 516	625	301, 463

* Anthocyanins were identified using HPLC retention times and UV and mass spectrometer data as compared with previously published data.

A change in anthocyanin profile was found in the petal tissue of the transgenic lines as might be expected with a reduction in *F3'5'H *activity (Figure. 6A). Delphinidin-derived (malvidin- or petunidin-based anthocyanins) pigment levels decreased as a proportion of the total anthocyanins in petal tissue of most of the transgenic lines while the proportion of cyanidin-derived pigments (peonidin- and cyanidin-based anthocyanins) increased. This shift in anthocyanin profile correlates with a loss of expression of the endogenous *CpF3'5'H *transcript (Figure. 3C). The greater the loss of expression, e.g. cv 'Purple' line #31685 and cv 'Wine-Red' line #31691, the greater the change in anthocyanin profile (Figure 6B). Pelargonidin, an anthocyanin pigment with a mono-hydroxylated B-ring, was not produced in the transgenic lines of either cultivar.

There was also a marked reduction in total anthocyanin concentration in petal tissue of the transgenic lines. Lines with modified flower colour showed a decrease in total anthocyanin concentration of up to 80% of that in untransformed controls (Figure. 6B). The difference in anthocyanin concentrations between the transgenic lines and their respective controls were statistically significant at the 5% level.

Flavonol profiles were also examined. Flavonols in the untransformed and transgenic lines were putatively identified as kaempferol and quercetin 3-glucosides, rutinosides and acylated rutinosides (data not shown). This is consistent with previous studies [2]. Total flavonol concentration in the transgenic lines showed a statistically significant increase in most lines (Table 2). The quercetin/kaempferol ratio also increased significantly in most transgenics lines of cv 'Purple' but decreased significantly in all the transgenic lines of cv 'Wine-Red' (Table 2).

**Table 2:** Flavonol concentration and ratios in petals of transgenic lines (mg.g.DW^-^^1^) (Mean ± SEM, n = 2).

Cultivar	Line	**Flavonols ± sem (mg.g.DW**^**-1**^**)**	Q/K ratio ± sem
'Purple'			
Regeneration	29006	2.7 ± 0.05	0.7 ± 0.05
	31704	1.6 ± 0.27	0.4 ± 0.06
Transgenic	31674	2.9^a ^± 0.06	1.3^a ^± 0.00
	31675	2.1 ± 0.05	0.6 ± 0.00
	31681	3.9^a ^± 0.07	2.0^a ^± 0.01
	31682	3.4^a ^± 0.12	1.2^a ^± 0.03
	31683	4.4^a ^± *	1.1^a ^± *
	31684	4.0^a ^± *	0.8 ± *
	31685	4.6^a ^± 0.03	1.6^a ^± 0.02
	31687	3.0^a ^± 0.12	1.5^a ^± 0.05
'Wine-Red'			
Regeneration	29009	2.1 ± 0.14	5.4 ± 0.01
	31706	1.7 ± *	4.4 ± *
Transgenic	31691	4.7^a ^± 0.30	1.3^a ^± 0.05
	31693	4.3^a ^± 0.54	1.5^a ^± 0.05
	31695	3.1^a ^± 0.02	1.9^a ^± 0.22
	31698	2.4 ± 0.13	3.1^a ^± 0.13

^a^values significantly different from the control at the 5% level

*For these samples n = 1

### Flower colour analysis

Expression of the introduced antisense *CpF3'5'H *transgene and resulting flavonoid concentration and profile changes in the transgenic lines were translated into visible flower colour changes (Figure. 4). Cultivar 'Purple' lines showed a loss of purple colour and became pink, while the cv 'Wine-Red' lines remained a similar pinkish hue but with reduced intensity (chroma).

The change in colour observed by eye was quantified by colour measurements using a colorimeter. The colour parameters, lightness [L], chroma [C] and hue angle [H°] were statistically significantly different from controls in lines with modified flower colour in most lines (Table 3). The exceptions were lines 31675 (cv 'Purple') and 31698 (cv 'Wine-Red') for L and C values. Both lines exhibited the least change in their anthocyanin profiles (Figures 6A, B). The majority of transgenic lines of both cultivars showed an increase in lightness (L) and a reduced intensity of colour (C). This is consistent with the decreased anthocyanin concentration in the petal tissue from the transgenic lines. There was also a clear shift in H° away from purple in the control line towards red in the transgenic lines of the 'Purple' cultivar. This change in hue angle correlates with a decrease in the proportion of delphinidin-derived anthocyanins. However line #31685, which had the largest proportion of cyanidin-derived pigments, did not have the largest shift in H°. Similarly the only line of the cv 'Wine-Red' transgenics showing a shift from delphinidin- to cyanidin-derived pigments (line #31691) did not show a significant change in H° while the other two transgenics did. The shift in hue angle for the cv 'Wine-Red' transgenics was in fact back to the purple region of the colour wheel. The overall shift, however, was very small and hue angle remained in the red/pink region.

**Table 3:** Flower colour characteristics for petal tissue of the control and transgenic lines.

Cultivar	Line	Colour parameters		
'Purple'		L	C	H°

Regeneration	31704	40 ± 0.5	71 ± 0.5	348 ± 0.9
Transgenic	31675	34 ± 0.3	69 ± 0.5	359^a ^± 0.6
Transgenic	31681	53^a ^± 6.9	67 ± 3.8	355^a ^± 1.8
Transgenic	31682	63^a ^± *	58^a ^± *	351 ± *
Transgenic	31683	55^a ^± 1.3	58^a ^± 0.9	1.2^a ^± 1.1
Transgenic	31684	64^a ^± 3.2	57^a ^± 3.4	352^a ^± 2.1
Transgenic	31685	57^a ^± 0.7	65^a ^± 1.5	359^a ^± 1.1
Transgenic	31687	59^a ^± 1.1	63^a ^± 1.4	355^a ^± 1.2
Transgenic	31674	66^a ^± 0.6	51^a ^± 0.7	5.1^a ^± 0.6

'Wine-Red'				

Regeneration	29009	38 ± 1.1	62 ± 0.5	1.7 ± 2.0
Transgenic	31691	63^a ^± 0.6	55^a ^± 1.2	359 ± 0.6
Transgenic	31695	65^a ^± 1.7	50^a ^± 1.4	357^a ^± 0.9
Transgenic	31698	45 ± 2.1	66 ± 0.8	357^a ^± 0.5

Colour parameters (lightness, chroma and hue angle, L, C, H°) were measured with a Minolta CR-200 tristimulus colorimeter. Lightness represents the proportion of total incident light that is reflected. Chroma is a measure of colour intensity in relative intensity units. Hue angle is derived from a CIELAB colour space wheel with values stepped counterclockwise from red/purple at 360°/0°, yellow at 90°, bluish-green at 180° and blue at 270° [36]. The values shown in the table are means of three petals from three flowers of each line and the SEMs.

^a^values significantly different from the control at the 5% level

*For these samples n = 1

## Discussion

Antisense suppression of *CpF3'5'H *was successful in changing anthocyanin profiles and flower colour in cyclamen. A shift from predominantly delphinidin-derived pigments to a greater relative proportion of cyanidin-derived pigments was achieved and in general this showed up as a concomitant shift in H°, the parameter indicating colour group. It is interesting that the degree of change in H° did not correlate with the degree of shift in pigments. The fact that the transformants also showed variable drops in total anthocyanin levels and changes in flavonol level and type illustrates both the links between the different pools of flavonoid substrates and the importance of the roles that anthocyanin concentration and flavonol copigmentation play in flower colour.

Similar changes in anthocyanin concentration and the accumulation of cyanidin-derived anthocyanins were seen for the two different minicyclamen cultivars and yet the greatest change in H° was seen in the lines of the purple cultivar. This is most likely due to a reduction in the predominant anthocyanin, malvidin 3-5-di-*O*-glucoside in these lines. This anthocyanin has been reported as being bluer in colour than malvidin mono-glucosides [3]. The predominant anthocyanin in the 'Wine-Red' cultivar is malvidin 3-*O*-glucoside and this has been reported to give pink/purple colours, closer to the colour associated with cyanidin and peonidin pigments [3].

Pelargonidin-based pigments were not detected in the flowers of the transgenics. One explanation for their absence is that suppression of F3'5'H activity was not complete, as evidenced by the presence of delphinidin-derived anthocyanins. This may be either due to inefficiency of the antisense approach (as opposed to hairpin RNA-induced RNAi [18]), effects due to transgene insertion or copy number [19-21], or the presence of other unaffected F3'5'H family members. The presence of a F3'H enzyme in the petals could have also removed substrate for pelargonidin production. We have searched for a cyclamen F3'H cDNA and found one (GenBank GU808358) with high deduced amino acid similarity to known *F3'H *sequences of other species (81% identity with the *F3'H *of gentian). However, transcript levels for this particular *F3'H *gene were not detectable by northern analysis during cyclamen petal development (unpublished data).

Substrate specificity is an important consideration regarding pelargonidin production. In some species, such as petunia [22], cymbidium [23,24] and *Osteospermum *[14], synthesis of pelargonidin-based anthocyanins is limited by the substrate specificity of the endogenous DFR. Our substrate feeding experiments (mentioned previously) showed that cyclamen has the ability to make pelargonidin-derived anthocyanins. It is still possible, however, that cyclamen DFR has low substrate specificity for DHK and the action of flavonol synthase (FLS), F3'H and F3'5'H means that the DHK substrate is not used for the synthesis of pelargonidin. Retransformation of an antisense F3'5'H line from this study, with a transgene encoding a DFR known to efficiently catalyse the reduction of DHK to leucopelargonidin [25-27] could result in transgenic plants accumulating pelargonidin derivatives in flowers, as successfully demonstrated for *Osteospermum *[14]. It remains to be resolved whether there is a F3'H functioning in the flower. The presence of cyanidin-based pigments in the flowers of the antisense *CpF3'5'H *lines suggests F3'H activity. Thus, inhibition of either F3'H or FLS gene activity to reduce enzymatic competition for DHK substrate may also be necessary to promote pelargonidin production in DFR/antisense F3'5'H transgenics.

In the cyclamen transgenic lines, total anthocyanin levels decreased markedly while flavonol levels increased and the quercetin/kaempferol ratio changed. Similar results were reported for *Nierembergia *flowers modified with an antisense *F3'5'H *construct and were suggested to be due to a modified flow through the flavonoid pathway [13]. A block in F3'5'H activity resulted in an increase in pelargonidin precursors. Low F3'H activity coupled with a DFR that putatively does not recognise DHK, was suggested to have led to limited substrate flow toward pigment production and an increase in the sustrate pool for FLS [13]. The flavonoid enzyme kinetics are not known for cyclamen. However, if the cyclamen DFR has a low specificity for pelargonidin or cyanidin precursors (as the reduction in total anthocyanins (Figure. 6B) suggests) this would provide extra substrate for the FLS enzyme and explain the increased flavonol levels. Competition for substrate between FLS and DFR has also been shown to occur in petunia [28,29].

It is interesting that while flavonol levels generally increased in the transgenics, there were differences in the quercetin/kaempferol ratios between the lines of the different cultivars. Quercetin flavonols increased in cv 'Purple' lines while kaempferol types increased in cv 'Wine-Red' lines. This inverse result and the consistency of the ratio change within lines of each cultivar argues against the suppression of F3'5'H activity directly altering the balance of DHK and DHQ, and thus what is available for the FLS. Furthermore, differing substrate specificities of their respective FLS cannot account for the observed results. Differing specificities of other enzymes are likely to be the cause. The probable candidate is F3'H, which in other species can not only alter the balance between DHK and DHQ, but also convert kaempferol to quercetin [30]. Further studies of cyclamen flower colour would warrant a continued search for a *F3'H*.

## Conclusions

We report here the first successful alteration of cyclamen anthocyanin pigmentation using genetic modification techniques. Our results highlight the intricate interplay between type and concentration of both anthocyanin pigments and flavonol co-pigments in flower colour and illustrate the complexity involved in modifying a biosynthetic pathway with multiple branch points to different end products.

## Methods

### Cloning of F3'5'H cDNA and sequence analysis

A cDNA library from mixed flower stages of *C. persicum *'Sierra Rose' petals was made using a lambda ZAPII bacteriophage vector kit (Stratagene, USA). This library was first screened with a heterologous clone of *F3'H *from petunia (Florigene Flowers, Australia) and a partial *F3'5'H *cDNA was found. The partial *F3'5'H *cDNA was used to rescreen the cDNA library to obtain a full length *CpF3'5'H *cDNA.

The MegAlign programme of Lasergene (DNASTAR Inc., Madison, USA) was used to compare the *CpF3'5'H *deduced amino acid sequence to ten known *F3'5'H *sequences (*Camellia sinensis *AAY23287; *Campanula medium *BAA03440; *Catharanthus roseus *CAA09850; *Eustoma grandiflorum *BAA03439; *Glycine max *ABQ96218; *Gossypium hirsutum *AAP31058; *Petunia hybrida *CAA80266; *Phalaenopsis hybrida *AAZ79451; *Solanum tuberosum *AAV85473; *Vitis vinifera *BAE47007), ten *F3'H *sequences (*Antirrhinum majus *ABB53383; *Arabidopsis thaliana *NP_196416; *Glycine max *ABW69386; *Ipomoea tricolor *BAD00192; *Matthiola incana *AAG49301; *Perilla frutescens *BAB59005; *Petunia hybrida *AAD56282; *Populus trichocarpa *XP_002319761; *Sorghum bicolor *ABG54321; *Vitis vinifera *ABH06586), cinnamate 4-hydroxylase from *Arabidopsis thaliana *(AAC99993) and flavone synthase II from *Medicago truncatula *(ABC86159).

### Construction of binary vectors

The *CpF3'5'H *cDNA was cloned into the *Eco*RI multiple cloning site of pART7 [31] in the antisense orientation to form pLN95. The *Not*I fragment from pLN95, which contains the 35S:antisenseF3'5'H:Ocs expression cassette, was ligated into the binary vectors; pART27 [31] to make pLN96, pMOA33 [32] to make pPN50, pMOA 34 [32] to make pPN51, and BJ49 [31] to make pPN48 (Figure. 3A). These binary vectors carried either the *npt*II or *hpt *selectable marker genes under a NOS promoter (Figure. 3A).

### Transformation with *Agrobacterium tumefaciens*

Etiolated hypocotyls of two parental lines of F1 hybrid minicyclamen cv 'Purple' and cv 'Wine-Red' were used as explants for transformation experiments. *A. tumefaciens *strain EHA105 containing either pLN96, pPN48, pPN50 or pPN51 were used to inoculate explants. The transformation protocol used was that reported by Boase *et al. *[15] except that hygromycin was used as the selection agent for cv 'Purple' lines using a range of concentrations: 5mg/l to day 12 after *Agrobacterium *inoculation, 20mg/l to day 77 after inoculation, then 15mg/l until shoots were recovered.

### Northern blot analyses

RNA was extracted from petal tissue for northern blot analysis using a modified hot borate method [33,34]. RNA was separated by electrophoresis on a 1% agarose RNA gel and subsequently transferred to Hybond XL nylon membranes using a SSC overnight blotting method. The membranes were hybridized with appropriate radioactively-labelled probes. The probe for *hpt *was a 1.1 kb *Xho*I fragment digested from pCAMBIA1301, which contained the *hpt *gene. The probe for *F3'5'H *was a 1.7 kb *Xba*I-*Eco*RI fragment digested from pLN95. Both membranes were also rehybrised to a cDNA probe corresponding to a 25/26S rRNA (pTip6) from *Asparagus officinalis, *to show RNA loadings. Autoradiography was conducted at -80°C using Kodak Biomax X-ray film.

### RT-PCR analysis of *nptII *mRNA transcripts

To investigate the expression of the introduced *npt*II selectable marker recombinant gene, RT-PCR analysis was performed on RNA extracted from petals using a modified hot borate method [33,34]. Three independent transgenic lines of cv 'Wine-Red' (#31691, #31695 and #31698) and one untransformed control (#29009) were tested. First strand cDNA was reverse transcribed from 100ngRNA per sample using Superscript II (Invitrogen USA) and oligo dT primer, and then 1 μl of the resulting cDNA per line was used for the PCR. For PCR, initial denaturation was at 94°C for 2 min followed by 40 cycles of melting (94°C/30 s), annealing (50°C/30 s) and extension (72°C/2 min). The *npt*II primers used were: forward 5'-ATGACTGGGCACAACAGACCATCGGCTGCT-3' and reverse, 5'-CGGGTAGCCAACGCTATGTCCTGATAGCGG-3'.

PCR products were separated electrophoretically on a 1% (w/v) NaB agarose gel stained with Sybr^®^safe (Invitrogen USA).

### Flavonoid analyses

Flavonoids were analysed by high performance liquid chromatography (HPLC) and liquid chromatography mass spectrometry (LC-MS). Freeze dried tissue was used for the analysis. Samples of ground freeze-dried petal tissue (50mg DW) were extracted initially in 2ml of methanol:acetic acid:water (70:3:27) and then re-extracted in 2 ml methanol:acetic acid:water (90:1:9). The combined supernatants were concentrated *in vacuo *and made up to a final volume of 1ml. HPLC analysis was carried out using a Waters 600 solvent delivery system with a Phenomenex Prodigy (5 μm, 250 × 4.6 mm) RP-18 endcapped column (column temperature 30°C) and a Waters 996 PDA detector. Solvent systems, flow rates and gradients are as described by Bloor *et al*. [35]. Flavonoids were detected at 350nm and anthocyanins at 530nm. Flavonoid levels were determined as quercetin-3-*O*-rhamnoglucoside (Apin Chemicals, Abingdon, Oxon, UK) equivalents, and the anthocyanins as cyanidin 3-*O*-glucoside (Extrasynthese, Genay, France) equivalents. Results are reported as the mean of the two replicates.

Separate extracts were analysed by electrospray mass spectrometry with a Thermo Finnigan LTQ ion-trap mass spectrometer. A Synergi Fusion RP80, 4 μm, 150 × 2.1 mm column with 4 × 2 mm guard cartridge from Phenomenex Ltd was used for separation. The mobile phase consisted of water (A) and acetonitrile (B) both containing 1% formic acid (FA). Extracts were injected at 5 μL volumes with a gradient program from 95% A to 50% A over 50 min. The column was washed by ramping to 90% B for 5 min and then re-equilibrated to the starting conditions for a further 5 min. Compound elution was monitored by PDA detector scanning the range 250-600 nm and by mass scanning from m/z 150-1500 to collect parent, MS^2 ^and MS^3 ^data in positive and negative ion (additional run) selection modes.

### Flower colorimeter analysis

Colours in all lines were quantified by measurement of three petals of each flower, three flowers per line with a Minolta CR-200 tristimulus colorimeter, set on CIELab D65 light source and 0° observer angle. Lightness (L) represents the proportion of total incident light that is reflected. Chroma (C) describes the degree to which selective absorption occurs i.e. colour saturation in relative intensity units. Hue angle (H) is derived from a CIELAB colour space wheel with values stepped counterclockwise from red at 0°/360°, yellow at 90°, bluish-green at 180° and blue at 270° [36].

### Statistics

A one-way ANOVA was performed on each data set shown in Tables 2 &3 and Figure 6B followed by a comparison of means using either a 5% Fisher's Least Significant Difference (5% LSD) to compare each line with a single control, or contrasts to compare each line with the combined mean of two controls. Lines with values significantly different from their control (or pair of controls) at the 5% level have been indicated by adding a superscript ^a ^to the means in Tables 2 and 3, and in Figure 6B. All analyses were performed using GenStat statistical software [37].

## Authors' information

Ms  Marshall and Ms Patel are former team members of the New Zealand Institute for Plant and Food Research Ltd.

## Authors' contributions

MRB designed and coordinated the experiments and analyses, carried out the genetic transformations, TLC analyses, colorimeter measurements, photography of phenotypes, and drafted the manuscript. DHL conducted the HPLC and UV absorption spectrophotometry analyses, arranged for the LC-MS analyses to be done, assisted with their interpretation, and helped draft the manuscript. KMD made the cDNA library and conducted the phylogenetic analysis. GBM isolated the *F3'5'H *cDNA, made the binary vector constructs, and electroporated them into the strains of *A. tumefaciens*. DP performed the northern analyses and did the RT-PCR with the *npt*II probe. KMD and KES conceived of the study, gave advice on molecular and biochemical analyses and helped draft the manuscript. SCD provided guidance on the molecular analyses and helped draft the manuscript. All authors read and approved the final manuscript.
